# Poly[bis­[μ_2_-1,4-bis­(1*H*-imidazol-1-yl)butane]­dichloridonickel(II)]

**DOI:** 10.1107/S1600536811044448

**Published:** 2011-10-29

**Authors:** Jia Zhang, Jiang-Feng Song

**Affiliations:** a Department of Chemistry, College of Science, North University of China, Taiyuan Shanxi 030051, People’s Republic of China

## Abstract

The asymmetric unit of the title compound, [NiCl_2_(C_10_H_14_N_4_)_2_]_*n*_, consists of one Ni^2+^ ion which is located on an inversion center, one 1,4-bis­(imidazol-1-yl)butane (bimb) and one chloride ion. The Ni^2+^ ion exhibits a distorted octa­hedral coordination environment defined by four N atoms from four bimb ligands in the equatorial plane and two chloride ions in axial positions. The bridging coordination mode of the bimb ligands leads to the formation of inter­penetrating square Ni_4_(bimb)_4_ units that are arranged parallel to (001). The separation between the Ni atoms in these units is 13.740 (3) Å.

## Related literature

For related structures based on bis­(imidazole)­alkane ligands, see: Ballester *et al.* (1998 ▶); Li *et al.* (2004 ▶); Zhu *et al.* (2006 ▶, 2009 ▶).
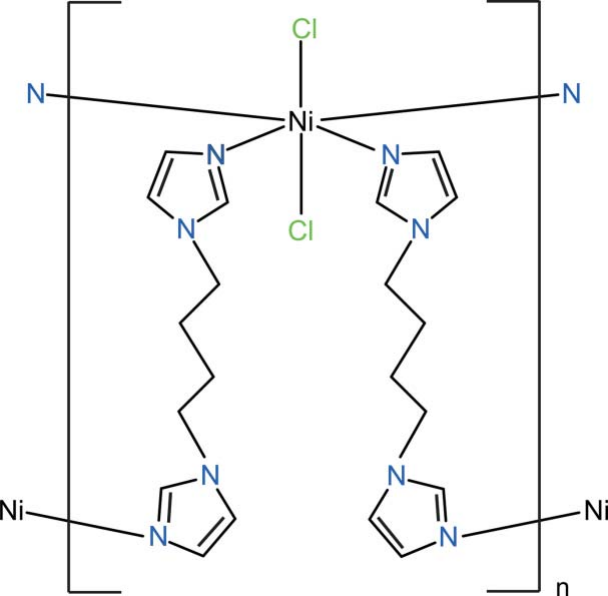


## Experimental

### 

#### Crystal data

[NiCl_2_(C_10_H_14_N_4_)_2_]
*M* *_r_* = 510.11
Monoclinic, 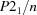

*a* = 7.4572 (15) Å
*b* = 18.297 (4) Å
*c* = 8.7321 (17) Å
β = 113.60 (3)°
*V* = 1091.8 (4) Å^3^
*Z* = 2
Mo *K*α radiation
μ = 1.16 mm^−1^
*T* = 293 K
0.20 × 0.15 × 0.10 mm

#### Data collection

Bruker SMART APEX CCD diffractometer
Absorption correction: multi-scan (*SADABS*; Bruker, 2004 ▶) *T*_min_ = 0.801, *T*_max_ = 0.893
10382 measured reflections
2469 independent reflections
2158 reflections with *I* > 2σ(*I*)
*R*_int_ = 0.037

#### Refinement

*R*[*F* ^2^ > 2σ(*F* ^2^)] = 0.037
*wR*(*F* ^2^) = 0.089
*S* = 1.11
2469 reflections
142 parameters
H-atom parameters constrained
Δρ_max_ = 0.33 e Å^−3^
Δρ_min_ = −0.37 e Å^−3^

### 

Data collection: *SMART* (Bruker, 2004 ▶); cell refinement: *SAINT* (Bruker, 2004 ▶); data reduction: *SAINT*; program(s) used to solve structure: *SHELXS97* (Sheldrick, 2008 ▶); program(s) used to refine structure: *SHELXL97* (Sheldrick, 2008 ▶); molecular graphics: *DIAMOND* (Brandenburg, 2000 ▶); software used to prepare material for publication: *SHELXL97*.

## Supplementary Material

Crystal structure: contains datablock(s) I, global. DOI: 10.1107/S1600536811044448/wm2543sup1.cif

Structure factors: contains datablock(s) I. DOI: 10.1107/S1600536811044448/wm2543Isup2.hkl

Additional supplementary materials:  crystallographic information; 3D view; checkCIF report

## Figures and Tables

**Table 1:** Selected bond lengths (Å)

Ni1—N4^i^	2.0980 (19)
Ni1—N1	2.111 (2)
Ni1—Cl1	2.5270 (8)

Symmetry code: (i) 
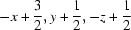
.

**Fig. 1.**
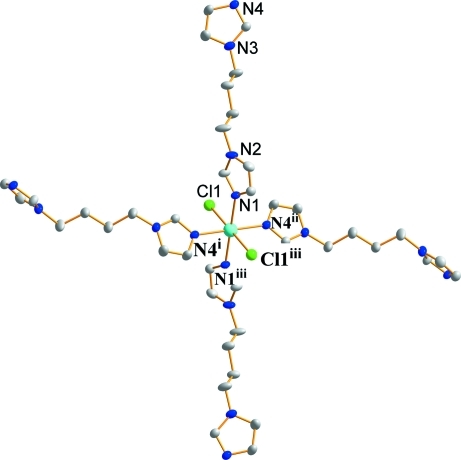
The coordination environment of Ni(II) atom with displacement ellipsoids at the 50% probability level. H atome were omitted for clarity. [Symmetry codes: i) 1.5-x, 0.5+y, 0.5-z; ii) 1.5+x, 0.5+y, 0.5+z; iii) 3-x, 1-y, 1-z]

**Fig. 2.**
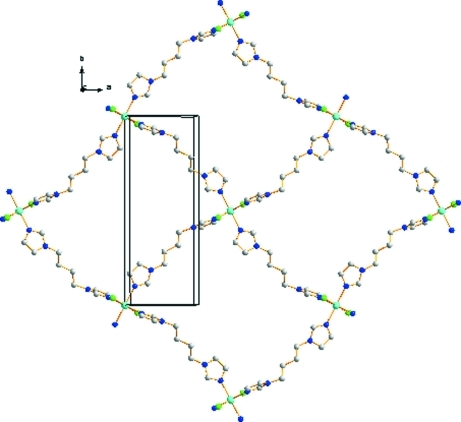
View of a two-dimensional layer containing square Ni4(bimb)4 units with dimension of 13.740 (3) × 13.740 (3) Å2 parallel to (001).

## supplementary crystallographic information

### Comment

A large number of novel topologies constructed from bis(pyridine)alkane, triazolealkane or bis(imidazole)alkane ligands have been reported in recent years, for example, [Co(bte)_2_(NCS)_2_]_n_, {[Cd(bte)_2_(H_2_O)_2_](NO_3_)_2_}_n_, or [Cd(bimb)_2_(NCO)_2_]_n_ [bte=1,2-Bis (1,2,4-triazol-1-yl) ethane, bimb=1,4-bis (imidazol-1-yl) butane] (Ballester *et al.*, 1998; Li *et al.*, 2004; Zhu *et al.*, 2006; Zhu *et al.*, 2009). These ligands show flexible bridging modes and can adopt different conformations (Li *et al.*, 2004). Herein, a new coordination polymer based on 1,4-bis(imidazol-1-yl)-butane, [Ni(bimb)_2_Cl_2_]_n_, is reported.

The asymmetric unit of the title compound consists of one Ni^2+^ ion which is located on an inversion center, one bimb ligand and one chloride ion. The Ni^2+^ ion exhibits a distorted octahedral coordination environment defined by four N atoms from four bimp ligands in the equatorial plane [Ni1—N4 = 2.0980 (19) Å; Ni1—N1 = 2.111 (2) Å] and two chloride ions in axial positions [Ni1—Cl1 = 2.5270 (8) Å]. The dihedral angle between the imidazole rings in the bimp ligand is 60.99 (16)°.

Each bimb ligand connects two adjacent Ni^2+^ ions to form interpenetrating two-dimensional networks containing square Ni_4_(bimb)_4_ units parallel to (001) (Figure 2). The square Ni_4_(bimb)_4_ units are constructed from four Ni^2+^ ions which are situated in the four corners and four bimb ligands which are in the edge positions. The Ni ··· Ni distance in the net is 13.740 (3) Å.

### Experimental

A solution of ethyl 2,2-difluoro-2-(pyridin-2-yl)acetate (10.0 mg, 0.05 mmol) in 2 ml ethanol was directly mixed with a solution of NiCl_2_ in 1 ml water (0.10 mol dm^-3^) at room temperature in a 15 ml beaker. A solution of bimb (9.5 mg, 0.05 mmol) in 3 ml e thanol in another 15 ml beaker was added the above-mentioned mixture. Then 2*M* HCl was added until the pH value of mixture arrives at 4.5. The resulted mixture was transferred and sealed in a 25 ml Teflon-lined stainless steel reactor, and heated at 85 °C for 72 h. Upon cooling to room temperature, the light green crystals were filtered and washed with water and ethanol.

### Refinement

All H atoms were located in a difference Fourier map refined as riding, with U_iso_(H) = 1.2U_eq_(C).

### Crystal data

**Table:**

[NiCl_2_(C_10_H_14_N_4_)_2_]	*F*(000) = 532
*M**_r_* = 510.11	*D*_x_ = 1.552 Mg m^−^^3^
Monoclinic, *P*2_1_/*n*	Mo *K*α radiation, λ = 0.71073 Å
Hall symbol: -P 2yn	Cell parameters from 2469 reflections
*a* = 7.4572 (15) Å	θ = 3.1–27.5°
*b* = 18.297 (4) Å	µ = 1.16 mm^−^^1^
*c* = 8.7321 (17) Å	*T* = 293 K
β = 113.60 (3)°	Block, green
*V* = 1091.8 (4) Å^3^	0.20 × 0.15 × 0.10 mm
*Z* = 2	

### Data collection

**Table:**

Bruker SMART APEX CCD diffractometer	2469 independent reflections
Radiation source: fine-focus sealed tube	2158 reflections with *I* > 2σ(*I*)
graphite	*R*_int_ = 0.037
ω scans	θ_max_ = 27.5°, θ_min_ = 3.1°
Absorption correction: multi-scan (*SADABS*; Bruker, 2004)	*h* = −9→9
*T*_min_ = 0.801, *T*_max_ = 0.893	*k* = −23→23
10382 measured reflections	*l* = −10→11

### Refinement

**Table:**

Refinement on *F*^2^	Primary atom site location: structure-invariant direct methods
Least-squares matrix: full	Secondary atom site location: difference Fourier map
*R*[*F*^2^ > 2σ(*F*^2^)] = 0.037	Hydrogen site location: inferred from neighbouring sites
*wR*(*F*^2^) = 0.089	H-atom parameters constrained
*S* = 1.11	*w* = 1/[σ^2^(*F*_o_^2^) + (0.0274*P*)^2^ + 1.1307*P*] where *P* = (*F*_o_^2^ + 2*F*_c_^2^)/3
2469 reflections	(Δ/σ)_max_ < 0.001
142 parameters	Δρ_max_ = 0.33 e Å^−^^3^
0 restraints	Δρ_min_ = −0.37 e Å^−^^3^

### Special details

**Table:**

Geometry. All e.s.d.'s (except the e.s.d. in the dihedral angle between two l.s. planes) are estimated using the full covariance matrix. The cell e.s.d.'s are taken into account individually in the estimation of e.s.d.'s in distances, angles and torsion angles; correlations between e.s.d.'s in cell parameters are only used when they are defined by crystal symmetry. An approximate (isotropic) treatment of cell e.s.d.'s is used for estimating e.s.d.'s involving l.s. planes.
Refinement. Refinement of *F*^2^ against ALL reflections. The weighted *R*-factor *wR* and goodness of fit *S* are based on *F*^2^, conventional *R*-factors *R* are based on *F*, with *F* set to zero for negative *F*^2^. The threshold expression of *F*^2^ > σ(*F*^2^) is used only for calculating *R*-factors(gt) *etc*. and is not relevant to the choice of reflections for refinement. *R*-factors based on *F*^2^ are statistically about twice as large as those based on *F*, and *R*- factors based on ALL data will be even larger.

### Fractional atomic coordinates and isotropic or equivalent isotropic displacement parameters (Å^2^)

**Table:**

	*x*	*y*	*z*	*U*_iso_*/*U*_eq_	
N4	0.1415 (3)	0.10177 (10)	0.0471 (3)	0.0289 (4)	
C9	0.0664 (4)	0.16648 (14)	0.0733 (4)	0.0440 (7)	
H9	−0.0615	0.1733	0.0630	0.053*	
C10	0.2054 (4)	0.21922 (14)	0.1165 (4)	0.0456 (7)	
H10	0.1917	0.2678	0.1415	0.055*	
C8	0.3245 (3)	0.11623 (13)	0.0740 (3)	0.0298 (5)	
H8	0.4118	0.0819	0.0650	0.036*	
N3	0.3700 (3)	0.18646 (11)	0.1160 (3)	0.0335 (5)	
C7	0.5640 (4)	0.21996 (15)	0.1668 (4)	0.0457 (7)	
H7A	0.6545	0.1835	0.1592	0.055*	
H7B	0.6092	0.2347	0.2829	0.055*	
C6	0.5694 (4)	0.28506 (14)	0.0646 (3)	0.0383 (6)	
H6A	0.4725	0.3204	0.0647	0.046*	
H6B	0.5361	0.2699	−0.0500	0.046*	
Ni1	1.5000	0.5000	0.5000	0.02315 (12)	
Cl1	1.30895 (8)	0.46277 (3)	0.66987 (8)	0.03495 (16)	
N1	1.2760 (3)	0.45639 (10)	0.2843 (2)	0.0269 (4)	
N2	0.9912 (3)	0.41380 (11)	0.1097 (3)	0.0323 (4)	
C3	1.0969 (3)	0.43850 (14)	0.2651 (3)	0.0329 (5)	
H3	1.0496	0.4425	0.3485	0.039*	
C1	1.2840 (4)	0.44240 (13)	0.1329 (3)	0.0320 (5)	
H1	1.3929	0.4500	0.1083	0.038*	
C2	1.1099 (4)	0.41598 (13)	0.0245 (3)	0.0355 (5)	
H2	1.0776	0.4021	−0.0859	0.043*	
C5	0.7692 (4)	0.32062 (14)	0.1322 (4)	0.0414 (6)	
H5A	0.8052	0.3315	0.2495	0.050*	
H5B	0.8631	0.2855	0.1253	0.050*	
C4	0.7874 (4)	0.38856 (16)	0.0476 (4)	0.0481 (7)	
H4A	0.7393	0.3799	−0.0717	0.058*	
H4B	0.7072	0.4264	0.0661	0.058*	

### Atomic displacement parameters (Å^2^)

**Table:**

	*U*^11^	*U*^22^	*U*^33^	*U*^12^	*U*^13^	*U*^23^
N4	0.0205 (10)	0.0275 (10)	0.0382 (11)	0.0007 (7)	0.0113 (9)	0.0027 (8)
C9	0.0290 (13)	0.0301 (13)	0.079 (2)	0.0015 (10)	0.0275 (14)	0.0015 (13)
C10	0.0374 (15)	0.0263 (12)	0.080 (2)	−0.0011 (10)	0.0303 (15)	−0.0013 (13)
C8	0.0236 (12)	0.0295 (11)	0.0370 (13)	0.0006 (9)	0.0128 (10)	0.0031 (9)
N3	0.0224 (10)	0.0295 (10)	0.0472 (12)	−0.0034 (8)	0.0126 (9)	0.0058 (9)
C7	0.0240 (13)	0.0422 (15)	0.0624 (18)	−0.0085 (11)	0.0085 (13)	0.0115 (13)
C6	0.0279 (13)	0.0359 (13)	0.0446 (14)	−0.0107 (10)	0.0078 (11)	0.0010 (11)
Ni1	0.01420 (19)	0.0230 (2)	0.0300 (2)	−0.00183 (14)	0.00640 (16)	−0.00287 (15)
Cl1	0.0247 (3)	0.0419 (3)	0.0393 (3)	−0.0057 (2)	0.0139 (3)	−0.0037 (2)
N1	0.0174 (9)	0.0279 (9)	0.0321 (10)	−0.0033 (7)	0.0066 (8)	−0.0038 (8)
N2	0.0238 (10)	0.0306 (10)	0.0337 (10)	−0.0097 (8)	0.0023 (9)	0.0023 (8)
C3	0.0225 (12)	0.0410 (13)	0.0327 (12)	−0.0079 (10)	0.0086 (10)	−0.0021 (10)
C1	0.0283 (13)	0.0310 (12)	0.0390 (13)	−0.0020 (9)	0.0157 (11)	−0.0035 (10)
C2	0.0390 (14)	0.0321 (12)	0.0305 (12)	−0.0053 (10)	0.0089 (11)	−0.0053 (10)
C5	0.0253 (13)	0.0344 (13)	0.0570 (17)	−0.0059 (10)	0.0086 (12)	0.0077 (12)
C4	0.0254 (13)	0.0481 (16)	0.0522 (16)	−0.0159 (11)	−0.0041 (12)	0.0121 (13)

### Geometric parameters (Å, °)

**Table:**

N4—C8	1.316 (3)	Ni1—N1^iv^	2.111 (2)
N4—C9	1.368 (3)	Ni1—N1	2.111 (2)
N4—Ni1^i^	2.0980 (19)	Ni1—Cl1	2.5270 (8)
C9—C10	1.355 (4)	Ni1—Cl1^iv^	2.5270 (8)
C9—H9	0.9300	N1—C3	1.319 (3)
C10—N3	1.368 (3)	N1—C1	1.371 (3)
C10—H10	0.9300	N2—C3	1.346 (3)
C8—N3	1.342 (3)	N2—C2	1.366 (3)
C8—H8	0.9300	N2—C4	1.469 (3)
N3—C7	1.467 (3)	C3—H3	0.9300
C7—C6	1.499 (4)	C1—C2	1.353 (3)
C7—H7A	0.9700	C1—H1	0.9300
C7—H7B	0.9700	C2—H2	0.9300
C6—C5	1.512 (3)	C5—C4	1.480 (4)
C6—H6A	0.9700	C5—H5A	0.9700
C6—H6B	0.9700	C5—H5B	0.9700
Ni1—N4^ii^	2.0980 (19)	C4—H4A	0.9700
Ni1—N4^iii^	2.0980 (19)	C4—H4B	0.9700
			
C8—N4—C9	105.1 (2)	N1^iv^—Ni1—Cl1	90.51 (6)
C8—N4—Ni1^i^	128.15 (16)	N1—Ni1—Cl1	89.49 (6)
C9—N4—Ni1^i^	126.43 (16)	N4^ii^—Ni1—Cl1^iv^	89.81 (6)
C10—C9—N4	110.2 (2)	N4^iii^—Ni1—Cl1^iv^	90.19 (6)
C10—C9—H9	124.9	N1^iv^—Ni1—Cl1^iv^	89.49 (6)
N4—C9—H9	124.9	N1—Ni1—Cl1^iv^	90.51 (6)
C9—C10—N3	105.9 (2)	Cl1—Ni1—Cl1^iv^	180.0
C9—C10—H10	127.0	C3—N1—C1	105.3 (2)
N3—C10—H10	127.0	C3—N1—Ni1	127.33 (17)
N4—C8—N3	111.9 (2)	C1—N1—Ni1	127.36 (15)
N4—C8—H8	124.1	C3—N2—C2	107.1 (2)
N3—C8—H8	124.1	C3—N2—C4	125.4 (2)
C8—N3—C10	106.9 (2)	C2—N2—C4	127.5 (2)
C8—N3—C7	126.4 (2)	N1—C3—N2	111.4 (2)
C10—N3—C7	126.4 (2)	N1—C3—H3	124.3
N3—C7—C6	114.2 (2)	N2—C3—H3	124.3
N3—C7—H7A	108.7	C2—C1—N1	110.0 (2)
C6—C7—H7A	108.7	C2—C1—H1	125.0
N3—C7—H7B	108.7	N1—C1—H1	125.0
C6—C7—H7B	108.7	C1—C2—N2	106.2 (2)
H7A—C7—H7B	107.6	C1—C2—H2	126.9
C7—C6—C5	111.5 (2)	N2—C2—H2	126.9
C7—C6—H6A	109.3	C4—C5—C6	116.1 (2)
C5—C6—H6A	109.3	C4—C5—H5A	108.3
C7—C6—H6B	109.3	C6—C5—H5A	108.3
C5—C6—H6B	109.3	C4—C5—H5B	108.3
H6A—C6—H6B	108.0	C6—C5—H5B	108.3
N4^ii^—Ni1—N4^iii^	180.0	H5A—C5—H5B	107.4
N4^ii^—Ni1—N1^iv^	90.26 (8)	N2—C4—C5	111.6 (2)
N4^iii^—Ni1—N1^iv^	89.74 (8)	N2—C4—H4A	109.3
N4^ii^—Ni1—N1	89.74 (8)	C5—C4—H4A	109.3
N4^iii^—Ni1—N1	90.26 (8)	N2—C4—H4B	109.3
N1^iv^—Ni1—N1	180.000 (1)	C5—C4—H4B	109.3
N4^ii^—Ni1—Cl1	90.19 (6)	H4A—C4—H4B	108.0
N4^iii^—Ni1—Cl1	89.81 (6)		

Symmetry codes: (i) −*x*+3/2, *y*−1/2, −*z*+1/2; (ii) *x*+3/2, −*y*+1/2, *z*+1/2; (iii) −*x*+3/2, *y*+1/2, −*z*+1/2; (iv) −*x*+3, −*y*+1, −*z*+1.
